# Photocatalytic Degradation of Wastewater by Molecularly Imprinted Ag_2_S-TiO_2_ with High-selectively

**DOI:** 10.1038/s41598-020-57925-8

**Published:** 2020-01-27

**Authors:** Xian Liu, Lei Zhu, Xun Wang, Xide Meng

**Affiliations:** School of Urban Construction, Wuhan University of Science and Technology, Wuhan, 430065 China

**Keywords:** Pollution remediation, Environmental impact, Photocatalysis

## Abstract

The molecular imprinting technique is a new method for preparing molecularly imprinted polymers (MIPs) with specific molecular recognition sites for individual target molecules. In this study, Ag_2_S-MIP-TiO_2_ nanocomposite was synthesized by a sol-gel-deposition method with ethyl p-hydroxybenzoate as an imprinting molecule. The obtained powder was characterized by XRD and other analytical methods. The results show that the obtained Ag_2_S-MIP-TiO_2_ nanocomposite demonstrates better catalytic performance than pure anatase TiO_2_. The degradation efficiency of ethyl p-hydroxybenzoate during 1.5 h of the photocatalytic reaction is 92.22%, which is 42% higher than pure TiO_2_. The selectivity factor for the treatment of ethyl p-hydroxybenzoate compared to phenol using Ag_2_S-MIP-TiO_2_ reached 3.571, which is 72% higher than pure TiO_2_.

## Introduction

Ethyl p-hydroxybenzoate (E-pHB) is widely used in the preservation of food and cosmetics for its antibacterial property, where it prevents microbial growth. Owing to broader usage of E-pHB, it has recently been detected in wastewater effluent. E-pHB is found resulting in long-term pollution to water environment, due to the difficulty of degradation^1^. Nano-TiO_2_ is environmentally friendly in removing E-pHB photocatalytically, however, the extent of degradation with TiO_2_ requires improvements by modification^2–5^.

Molecular imprinting technology is a technique for obtaining Molecular Imprinted Polymers (MIPs) which creates the same spatial structure and binding site and perfectly matches the target molecule^6^. Dickey first proposed the term “molecular imprinting” in 1949^7^. The German Wulff research team first synthesized highly selective MIPs in 1972, making molecular imprinting technology a research hotspot^8^. Deng *et al*. successfully synthesized MIP-PPy/TiO_2_ by a sol-gel method where methyl orange was employed as a template molecule^9^. The first-order k value of the molecularly imprinted TiO_2_ for the degradation of methyl orange was 2.3 times higher compared to pure TiO_2_. Wei Shengpei *et al*. used surface molecular imprinting technique to prepare nitrogen-doped TiO_2_ molecularly imprinted polymer^10^. It was found that nitrogen doping can cause a red shift of the light absorption band of the material with good selectivity. Thus, combining molecular imprinting technology with nano-TiO_2_ can significantly improve the photocatalytic activity of pure TiO_2,_ thereby enhancing its capability of treating wastewater selectively^11,12^.

Silver sulfide (Ag_2_S) is another narrow band semiconductor material with broad application prospects in multiple fields as antibacterial/microbial agent and photocatalysis^13,14^. In this study, Ag_2_S as a hybrid material was added to MIP-TiO_2_ to improve its photocatalytic activity and to treat wastewater selectively. Overall, this investigation provided a new strategy for the preparation of traditional sol-gel based TiO_2_ photocatalytic materials to increase the selectivity and photocatalytic activity of TiO_2_ significantly.

## Results and Discussion

### XPS and XRD characterization

**Figure 1:**
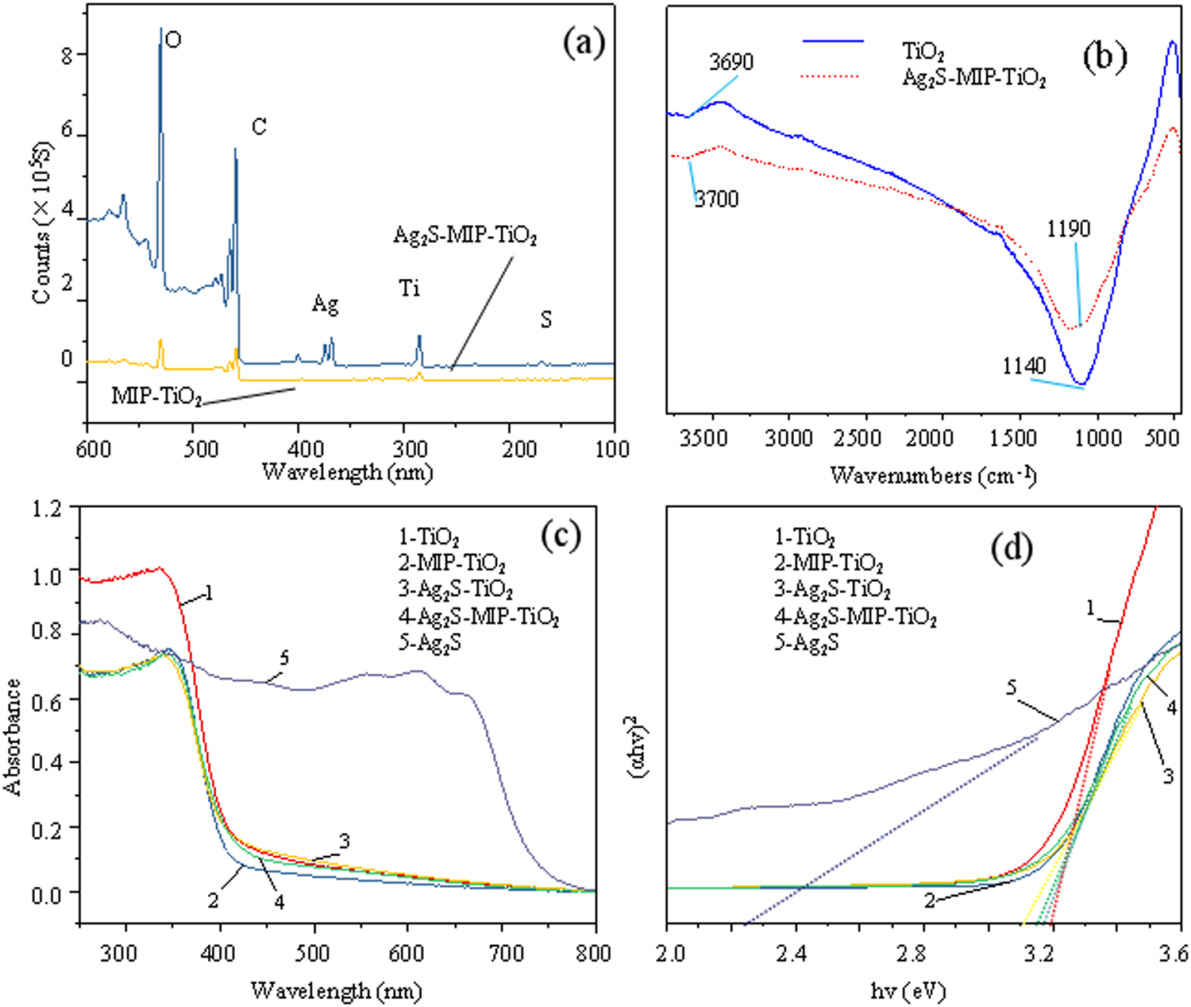
(**a**)- Full spectrum of XPS spectra of MIP-TiO_2_ and Ag_2_S-MIP-TiO_2_; (**b**)-FT-IR diagram of TiO_2_ and Ag_2_S-MIP-TiO_2_; (**c**)-UV-vis diagram of different catalysts; (**d**)-(αhv)^2^-hv curv.

The full spectrum of XPS on TiO_2_ and Ag_2_S-MIP-TiO_2_ samples are shown in Fig. 1(a). The peak positions of TiO_2_ correspond to the orbits of O1s, Ti2p, and C1. The characteristic peaks of Ag and S elements appeared in the spectrum of Ag_2_S-MIP-TiO_2_, which are derived from the doping of silver nitrate and thiourea. The shape of the peak of Ag_2_S-MIP-TiO_2_ is more than the absorption peak of Ag and S as compared to pure TiO_2_, indicating that silver and sulfur element are successfully doped into Ag_2_S-MIP-TiO_2_. It can be seen from Table 1 that a considerable amount of carbon element appears in the XPS spectrum of the two catalysts, which is due to the artificial addition for the calibration of the instrument during the test. The area ratio of Ti:Ag:S of Ag_2_S-MIP-TiO_2_ is 1:0.23:0.02, and the atomic ratio is 1:0.06:0.07, which is the same as that of the doping ratio, but the ratio of silver and sulfur element is too large. This might be due to the increase in the error caused by the sample itself and the excessive doping of Ag and S. It can be seen from Table 1 that the atomic ratio of Ti:O is 2.1:1, indicating that the main component is TiO_2_.

**Table 1:** XPS elemental analysis table of catalyst.

catalyst	elements	Start BE	Peak BE	End BE	FWHM eV	Area (P) CPS.eV	Atomic %
Ag_2_S-MIP-TiO_2_	S2p	174.95	167.97	157.05	2.03	6608.35	1.72
	C1s	301.95	284.15	278.05	1.44	45300.52	25.32
	Ag3d	379.95	367.46	360.05	1.1	66234.99	1.55
	O1s	544.95	529.43	525.05	1.24	254101.56	48.44
	Ti2p	474.95	458.18	448.05	1.08	279102.28	23.06
MIP-TiO_2_	C1s	301.95	284.09	278.05	1.32	8700.97	28.8
	Ti2p	474.95	457.95	448.05	1.05	49891.49	27.01
	O1s	534.9	529.16	525.05	1.18	35382.6	44.2

Ag_2_S can be obtained by the chemical reaction of thiourea and silver nitrate. In this study, the effect of nitrogen element in thiourea is neglected as in the gel drying and calcination stage, 1% of nitrogen element is extremely volatile so that the catalyst synthesized is a Ag_2_S-MIP-TiO_2_. The main reactions involved are as follows:

1 $$2AgN{O}_{3}+CS{(N{H}_{2})}_{2}=A{g}_{2}S\downarrow +CO{(N{H}_{2})}_{2}+HN{O}_{3}$$

2 $$CO{(N{H}_{2})}_{2}+{H}_{2}O\mathop{=}\limits^{\Delta }N{H}_{3}\uparrow +C{O}_{2}\uparrow $$

3 $$4HN{O}_{3}\mathop{=}\limits^{\Delta }4N{O}_{2}\uparrow +{O}_{2}\uparrow +2{H}_{2}O$$

4 $${C}_{9}{H}_{10}{O}_{3}+A{g}_{2}S+Ti{O}_{2}\mathop{\to }\limits^{\Delta 500^\circ C}A{g}_{2}S-MIP-Ti{O}_{2}+C{O}_{2}\uparrow +{H}_{2}O\uparrow $$

**Figure 2:**
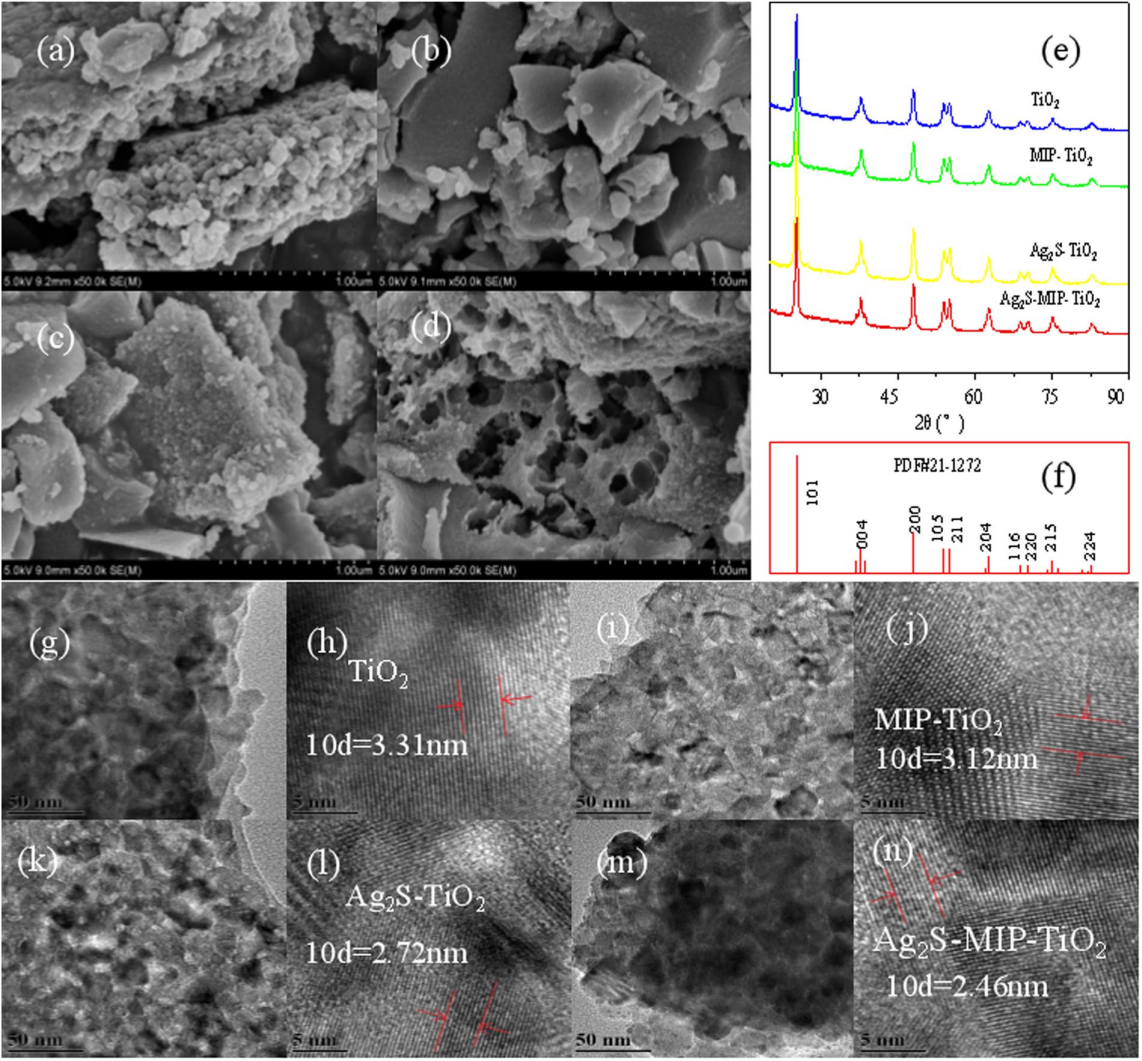
SEM image of (**a**)-TiO_2_, (**b**)-MIP-TiO_2_, (**c**)-Ag_2_S-TiO_2_, (**d**)-Ag_2_S-MIP-TiO_2_; (**e**)-XRD pattern of different catalysts, (**f**)-PDF#21–1272; TEM image of (**g**,**h**)-TiO_2_, (**i**,**j**)-MIP-TiO_2_, (**k**,**l**)-Ag_2_S-TiO_2_, (**m**,**n**)-Ag_2_S-MIP-TiO_2_.

Figure 2(e) shows the XRD patterns of TiO_2_, MIP-TiO_2_, Ag_2_S-TiO_2_, and Ag_2_S-MIP-TiO_2_ samples. The four samples demonstrated strong diffraction peaks at 25.281°, 37.8°, and 48.049°, which correspond to the crystal planes of (1-0-1), (0-0-4), (2-0-0), etc. of the standard diffraction card of anatase TiO_2_ (PDF#21-1272). No characteristic peaks from the (2-1-1) crystal plane of brookite and (1-1-0) crystal plane of rutile indicated the presence of anatase crystals. The characteristic peak of (1-1-0) crystal plane of Ag_2_S was noted at 25.726°, which was covered by strong diffraction peaks in the vicinity of (1-0-1) crystal plane of anatase TiO_2_ at 25.281°. Therefore, the addition of Ag_2_S did not affect the crystallization of TiO_2_. The average particle diameters of TiO_2_, MIP-TiO_2_, Ag_2_S-TiO_2_, and Ag_2_S-MIP-TiO_2_ were determined by X-ray diffraction line width method (Scherrer formula) as 16.7, 16.1, 15.5, and 15.4 nm respectively^15^. It indicates that the addition of Ag_2_S or imprinted molecules could reduce the particle size of TiO_2_. TiO_2_ with a particle size of less than approximately 25 nm has a significant quantum size effect so that Ag_2_S-MIP-TiO_2_ can provide better photocatalytic activity^16^.

### UV-Vis characterization

As shown in Fig. 1(d), the absorption of ultraviolet light by MIP-TiO_2_, Ag_2_S-TiO_2_, and Ag_2_S-MIP-TiO_2_ is reduced as compared to TiO_2_, and the utilization of ultraviolet light is lowered, which reduce the activity of the materials. The absorption of Ag_2_S at 400–700 nm indicates the ability of Ag_2_S to absorb the visible light. The band structure of catalyst was investigated by the direct bandgap semiconductor light absorption edge equation as shown below^17^.

5 $$\alpha {\rm{hv}}={\rm{A}}{({\rm{hv}}-{\rm{Eg}})}^{1/2}$$

Where α is the value of light absorption (abs), Hv is the incident light energy (eV), A and Eg are constants, and Eg represents the forbidden bandwidth (eV).

Figure 1(d) was obtained using UV-vis data and Eq. 5. A linear fit was performed with the data around 3.2 eV, and the intercept of the transverse axis of different fitted lines was obtained as the indirect forbidden band width of the powder. As shown in Table 2, the forbidden bandwidths of TiO_2_, MIP-TiO_2_, Ag_2_S-TiO_2_, Ag_2_S-MIP-TiO_2_, and Ag_2_S, in turn, decreased from 3.2 to 2.28 eV. This suggested that the addition of Ag_2_S reduced the forbidden bandwidth of Ag_2_S-TiO_2_ and Ag_2_S-MIP-TiO_2_, which formed a new energy level enabling the capturing of ultraviolet light with lower energy.

**Table 2:** Forbidden band of different catalysts.

samples	TiO_2_	MIP-TiO_2_	Ag_2_S-TiO_2_	Ag_2_S-MIP-TiO_2_	Ag_2_S
Eg/eV	3.2	3.18	3.15	3.13	2.28

As the size of the crystallites of semiconductor TiO_2_ was reduced to a certain extent, the electron energy level near the Fermi level changes from quasi-continuous to splitting energy level, which makes the energy gap of the material wider. As the particle size of TiO_2_ decreases, the bandgap becomes wider. In the UV-Vis, the absorption peak can be seen to move in the short wavelength direction, i.e., the blue shift phenomenon. The presence of enabling level is reduced, when the electron confinement moves in a small volume, the particle size is reduced such that the electron wave function overlaps, there is an additional energy level such that the energy level spacing of the electron transition is reduced, the external force enabling gap is reduced, or there are vacancy and impurities. The reduction of the enabling level can cause the absorption peak to move toward the long wavelength, i.e., the red shift phenomenon on the UV-Vis. The blue and red shifts can also be explained by the following Eq. 6^18^:

6 $$E(r)={E}_{g}+\frac{{\pi }^{2}{h}^{2}}{2\mu {r}^{2}}-\frac{1.78{e}^{2}}{\varepsilon r}-0.248{E}_{Ry}^{\ast }$$

where, E(r) represents an absorption bandgap, Eg is a constant, r is the radius of the particle, μ represents the reduced mass of the particles, e represents the energy of the electron and ERy* indicates the effective Rydberg quantity.

The second item, (π^2^h^2^)/(2μr^2^), represents the amount of blue shift, and the third item, (1.78e^2^)/(εr), represents the amount of red shift. It can be noted that the interaction of the two causes the blue or red shift of the absorption peak on UV-Vis. When the amount of blue shift is higher than the red shift, it is possible to make the absorption peak as blue shift. Therefore, with the introduction of Ag_2_S, the phenomenon of the red shift may not also be observed.

### FT-IR characterization

Figure 1(b) shows the FT-IR spectra of TiO_2_ and Ag_2_S-MIP-TiO_2_, which exhibit absorption peaks around 1100 and 3700 cm^−1^. The absorption peak near 1190 cm^−1^ could be ascribed to the bending vibration of the O-H bond of the crystal water^19^. The peak around 3700 cm^−1^ is due to the free hydroxyl group. Due to MIP, the light absorption intensity of Ag_2_S-MIP-TiO_2_ at 3700 cm^−1^ became stronger, demonstrating that MIP assists the catalyst in forming stronger intermolecular forces with target contaminating molecules. In this figure, no absorption peak could be noted between 1750 and 1735 cm^−1^ arising from -C=O-, as well as an absorption peak of benzene ring skeleton between 1625 and 1450 cm^−1^, indicating that the imprinted molecule is completely eluted. After complete elution, the template molecule left the imprinted cavity on Ag_2_S-MIP-TiO_2_, which increased its ability to adsorb and identify target contaminants selectively, making TiO_2_ have higher catalytic activity and selective ability to remove E-pHB.

### SEM and TEM observations

As shown in Fig. 2(a–d), the morphology of all four samples show a specific size in the nm range, which confirm that the solution could render the Tyndall effect in the precursor phase^20^. It could be seen from Fig. 2(a) that TiO_2_ is well dispersed. The addition of Ag_2_S allows particles to disperse better (Fig. 2(c)), and when compare to pure TiO_2_ particles, they are smaller in size. Bubble-like nanoholes could be noted in some parts of the SEM image of Ag_2_S-MIP-TiO_2_ which may be due to the easy agglomeration of Ag_2_S^21^, leading to the agglomeration of imprinted molecules, and leaving an imprinted cavity of nanoscale after elution at high temperatures. In summary, the porous structure of Ag_2_S-MIP-TiO_2_ making it lighter in density and weight, and is more capable of capturing target contaminants.

Figure 2(e,f) shows the TEM images of the four samples. From the left side of the image, it could be noted that the crystal surfaces of all four samples are blurred and do not show any apparent isotropic growth states. The lattice stripes on the right side are clear and tidy, indicating the presence of a reasonable degree of crystallization. The grain sizes of TiO_2_, MIP-TiO_2_, Ag_2_S-TiO_2_, and Ag_2_S-MIP-TiO_2_ were estimated to be 19–29 nm, 18–34 nm, 13–24 nm, and 13–25 nm, respectively using ImageJ software. The lattice spacing of TiO_2_, MIP-TiO_2_, Ag_2_S-TiO_2_, and Ag_2_S-MIP-TiO_2_ are 0.33 nm, 0.31 nm, 0.27 nm, and 0.25 nm, respectively. The pitch corresponds to the pitch of the (101) crystal plane of the main diffraction peak. It can be seen that the addition of Ag_2_S causes TiO_2_ photocatalyst to have a reduced grain size with better crystallinity.

### BET characterization

**Figure 3:**
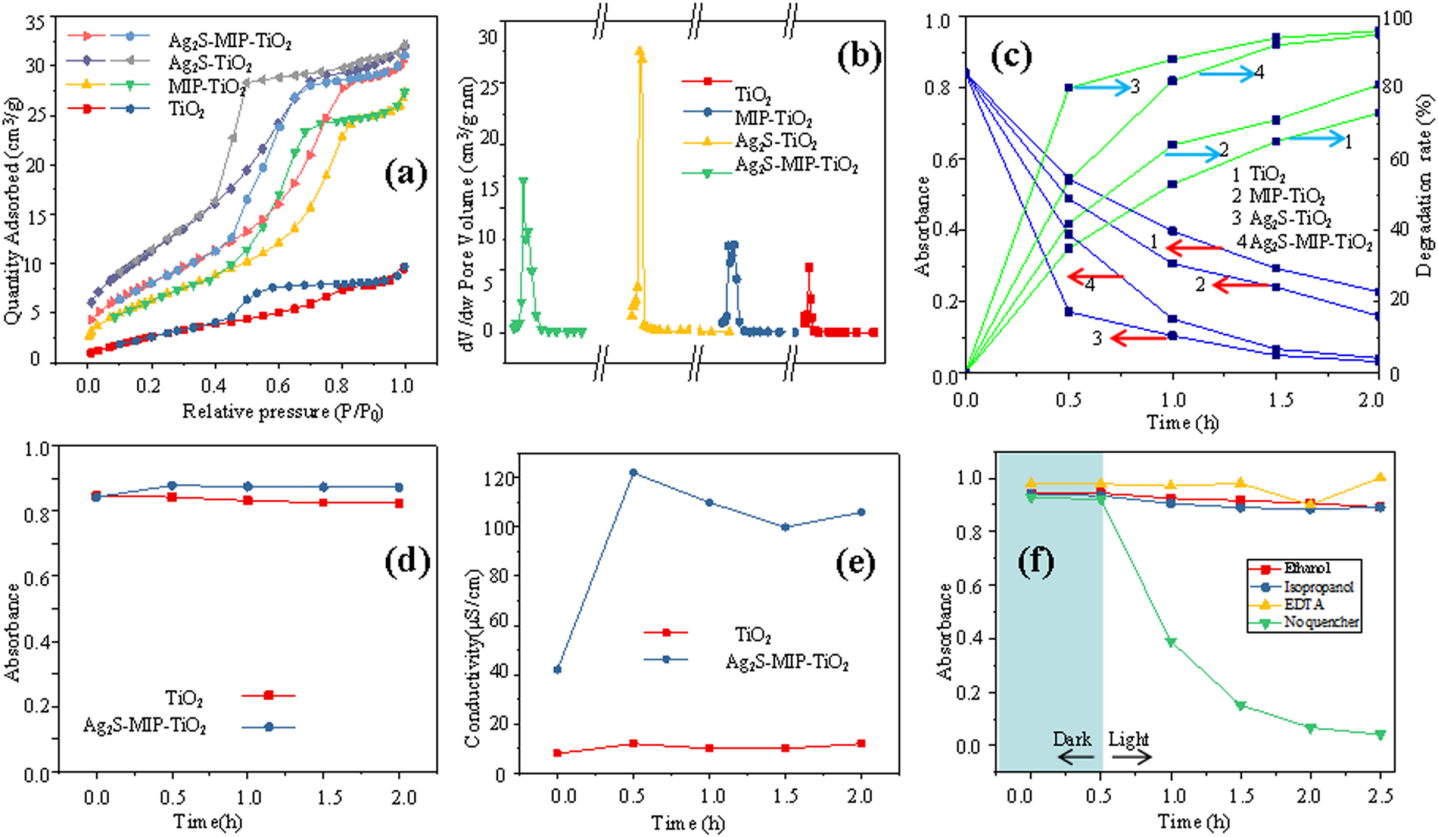
(**a**)-N_2_ adsorption desorption curve; (**b**)-BJH pore size distribution (0–20 nm); (**c**)-Comparison of photocatalytic activity of the catalyst; (**d**)-Dark reaction adsorption performance of the catalyst; (**e**)-Change in conductivity of the reaction solution; (**f**)-Effect of quencher on photocatalysis.

Figure 3(a) shows the N_2_ adsorption-desorption curve and pore size distribution of different catalysts. The four powders demonstrated distinct adsorption of H3 hysteresis loopback, which proved the existence of mesopores^22^. According to IUPAC’s definition of mesoporous materials, the four materials revealed adsorption hysteresis loops at a relative pressure between 0.4 and 0.8, which is consistent with the Langmuir IV curve, indicating that the materials are mesoporous adsorbents. As shown in Fig. 3(b), the pore sizes of the four materials are similar, ranging from 2 to 20 nm, which is consistent with the mesoporous nature of the material. The specific surface areas of TiO_2_, MIP-TiO_2_, Ag_2_S-TiO_2_, and Ag_2_S-MIP-TiO_2_ were 9.75, 24.79, 39.80, and 28.93 m^2^/g, respectively; and the average pore diameters were 3.92, 5.69, 3.56, and 4.51 nm, respectively. The following equation 7 was used to investigate the effect of specific surface area on the catalytic activity^23^.

7 $$\Sigma =S\cdot {\rho }_{0}\delta $$

Where Σ is the surface atomic fraction of a solid, surface effect; S is the specific surface area (m^2^/g), and ρ_0_ is the skeletal density (g/cm³), where all the four samples were considered to be 1 g/cm³, δ is the atomic spacing (nm), which is replaced by the average lattice spacing for the ease of comparison.

By comparing all four powders, it could be noted that the larger the specific surface area, the stronger the surface effect. The specific surface area of Ag_2_S-MIP-TiO_2_ is 2.97 times higher as compared to TiO_2_, and the surface effect is 2.25 times higher as compared to TiO_2_. The surface effect of Ag_2_S-TiO_2_ was observed to be the strongest, indicating that the smaller the grain size, the higher the surface energy and the stronger the photocatalytic activity, which is consistent with the results of XRD analysis. Since Ag_2_S-MIP-TiO_2_ showed smaller particle size than TiO_2_, it has more atoms on the surface and larger specific surface area, which improved the ability of Ag_2_S-MIP-TiO_2_ to capture pollutant molecules, thereby enhanced the photocatalytic ability to degrade organic matter.

### Analysis of photocatalytic degradation

As shown in Fig. 3(c), Ag_2_S-MIP-TiO_2_ and Ag_2_S-TiO_2_ demonstrate noticeable degradation effects on E-pHB, and the absorbance dropped within the first 30 min. The degradation effect increased with an increase in the illumination time, and the degradation rate could reach about 90% at 1 h after the reaction started, which is much higher than the degradation effect of MIP-TiO_2_ and TiO_2_. It indicates that Ag_2_S-MIP-TiO_2_ has a higher capability to degrade E-pHB and hence a higher catalytic activity. In Fig. 3(c), the photocatalytic performance of Ag_2_S-MIP-TiO_2_ is lower than Ag_2_S-TiO_2_. This may be due to the elution of the imprinted template molecules when MIP-TiO_2_ is prepared, which causes the catalyst itself to be polluting and is not conducive to rapid dispose of targeted pollutant wastewater. Molecular imprinting may hinder the process of light absorption of TiO_2_, thereby reducing the photocatalytic activity of MIP-TiO_2_ greatly. The selectivity and activity of MIP-TiO_2_ may interact with each other, sometimes increasing the selectivity of TiO_2_ but reducing its catalytic activity.

The reason for the selection of phenol is that both the structure of phenol and ethyl p-hydroxybenzoate possesses a benzene ring and a hydroxyl group on the benzene ring. The comparison of similar contaminants more reasonably reflects the selectivity, and thus phenol was used for selective studies. The selectivity factor of the target photo contaminant by the imprinted photocatalyst was obtained by analyzing the kinetic parameters of the degradation reaction^24^. As shown in Table 3, the selectivity factor, α of Ag_2_S-MIP-TiO_2_ is 3.571, which is 1.73 times compared to MIP-TiO_2_. It could be seen that the addition of Ag_2_S improved the photocatalytic activity and selectivity of MIP-TiO_2_. This could be due to the contribution of Ag_2_S to a certain partial agglomeration on the dispersion of the imprinted molecules in the precursor stage of TiO_2_, which formed a partially dispersed nano-imprinted cavity, and provided a binding site for the interaction with target contaminants. These binding sites adsorb and encapsulate target contaminants in the nanoscale imprinted cavity. Hence, the selectivity of Ag_2_S-MIP-TiO_2_ was improved.

**Table 3:** Comparison of reaction kinetic parameters.

Pollutants	catalyst	K	R(K_1_/K_2_)	α(Rtarget/Rnon-target)
E-pHB	MIP-TiO_2_	0.818	1.284	2.068
	TiO_2_	0.637		
phenol	MIP-TiO_2_	0.211	0.621	
	TiO_2_	0.34		
E-pHB	Ag_2_S-TiO_2_	2.316	3.636	1.449
	TiO_2_	0.637		
phenol	Ag_2_S-TiO_2_	0.853	2.509	
	TiO_2_	0.34		
E-pHB	Ag_2_S-MIP-TiO_2_	2.274	0.982	3.571
	Ag_2_S-TiO_2_	2.316		
phenol	Ag_2_S-MIP-TiO_2_	0.235	0.275	
	Ag_2_S-TiO_2_	0.853		

**Figure 4:**
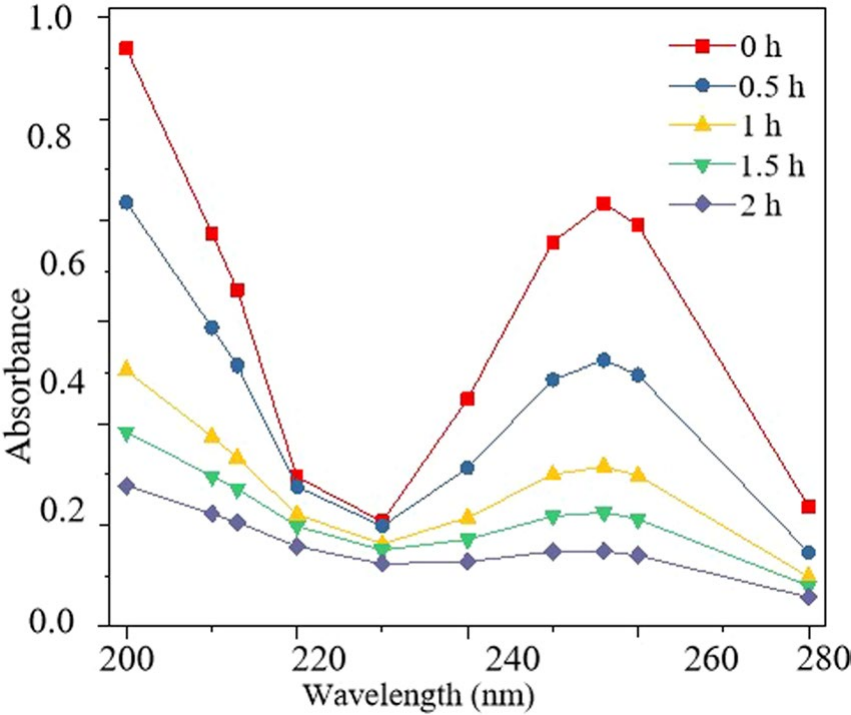
Multi-wavelength measurement of Ag_2_S-MIP-TiO_2_ processing E-pHB solution.

From Fig. 4, the two absorption peaks of E-pHB could be observed at 213 nm and 256 nm, which correspond to the characteristic absorption peaks of –OH and –COOC_3_H_7_ groups on the benzene ring, respectively. When E-pHB was treated, the rate of decrease in the absorbance of the stock solution at the wavelength of 213 nm is much lower than the rate of decrease in the absorbance at 256 nm, confirming that in the degradation of E-pHB by Ag_2_S-MIP-TiO_2_, the –OH group were degraded followed by the –COOC_3_H_7_ group.

To distinguish the main active substances of the reaction, an active material quenching experiment was carried out. Both isopropanol and ethanol are quenchers of •OH, which have a reaction rate constant of 1.9 × 10^9^ with •OH^25–27^, while ethylenediaminetetraacetic acid (EDTA) is a widely accepted capturing agent for holes^28^. 0.1 mol·L^−1^ of ethanol, isopropanol, and EDTA were added to the reaction solution to quench the hydroxyl radicals and photogenerated hole, which might be generated by the photocatalytic reaction. Figure 3(f) reflects the absorbance versus time curve of the reaction solution of Ag_2_S-MIP-TiO_2_ under each control experiment. It could be seen from this figure that in the reaction solution with ethanol, isopropanol, and EDTA, the residual ratio of ethyl p-hydroxybenzoate is above 90%, indicating that both hydroxyl radicals and holes demonstrate a significant influence on the photocatalytic reaction.

**Figure 5:**
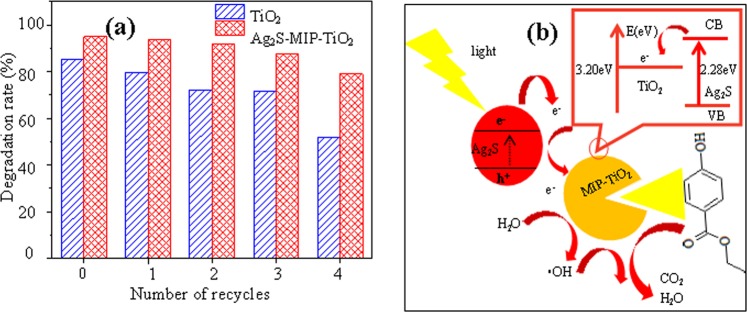
(**a**)-Comparison of recycalability of Ag_2_S-MIP-TiO_2_ and TiO_2_; (**b**)-Principle of photocatalysis of Ag_2_S-MIP-TiO_2_.

The mechanism of the photocatalytic degradation of organic pollutants by Ag_2_S-MIP-TiO_2_ is shown in Fig. 5(b). Under the irradiation of the ultraviolet light source, Ag_2_S generates photo-generated carriers, and MIP-TiO_2_ could identify the target pollutants selectively. The synergistic effect of the two catalysts causes increased electron transport efficiency of Ag_2_S-MIP-TiO_2_ and the recombination efficiency of electrons and holes decreased. Further, •OH was generated from the photogenerated carriers, and the organic matter was finally oxidized to CO_2_ and H_2_O^29,30^.

### Adsorption and conductivity analysis

To investigate the adsorption performance of the material, an isotherm adsorption experiment under dark conditions was carried out before the photocatalytic reaction. The obtained results are shown in Fig. 3(d), and it could be noted that pure TiO_2_ and Ag_2_S-MIP-TiO_2_ reached the adsorption equilibrium after 30 min, and the dark adsorption effect was poor, and even negative adsorption occurred. This could be due to the weak adsorption performance of the catalyst, and a small amount of soluble impurities lead to the increase of absorbance. An excessive adsorption performance might result in the catalyst being encapsulated by organic matter, which is not conducive to the continuous reaction. The weak and rapid adsorption performance was beneficial to the continuous and efficient photocatalytic reaction of the catalyst. Under the constant conditions of reaction pH and temperature, the physical adsorption and chemisorption of E-pHB by pure TiO_2_ and Ag_2_S-MIP-TiO_2_ were not strong, and the adsorption effect was negligible so that the dark adsorption experiment was not carried out subsequently.

To examine the overall changes of the photocatalytic reaction system, the change in the conductivity of the reaction system was monitored before and after the photocatalytic reaction. The obtained results are shown in Fig. 3(e). The conductivity of TiO_2_ and Ag_2_S-TiO_2_ mixed with the reaction solution was 10 and 42 μS/cm, respectively, indicating that the addition of Ag_2_S increased the conductivity of the reaction system. After 0.5 h of photoreaction, the conductivity of Ag_2_S-TiO_2_ reaction solution increased to 120 μS/cm and stabilized gradually, while the conductivity of TiO_2_ reaction solution always changed smoothly. In the reaction solution, only Ag_2_S-TiO_2_, water, and very little E-pHB were added; these substances did not affect changing the conductivity directly within 0.5 h of photoreaction. It indicates that a large amount of conductive materials was formed in the reaction solution within 0.5 h of photoreaction. The photogenerated carriers and ·OH generated by photoreaction increased the conductivity of the reaction solution. Since the amount of catalyst was constant, the subsequent conductivity was stabilized gradually. The conductivity of Ag_2_S-TiO_2_ reaction solution was higher than that of pure TiO_2_ reaction solution, indicating that the addition of Ag_2_S could change the conductivity of the photocatalytic reaction solution, thereby increasing the transfer rate of photogenerated carriers.

### Recyclability study

Figure 5(a) displays the recovery times with the corresponding degradation rate of 2 h. After four recovery cycles, the degradation rate of pollutants gradually decreased, but the rate remained above 80%. Compared with the recovery rate of pure TiO_2_ under the same conditions, the degradation rate of Ag_2_S-MIP-TiO_2_ powder was always higher than pure TiO_2_, and its declining rate was always lower than pure TiO_2_. This revealed that Ag_2_S-MIP-TiO_2_ powder not only had higher photocatalytic activity, but also had excellent recyclability, and its relative recycle rate to pure TiO_2_ was enhanced. Moreover, higher recyclability not only reflected the nature of Ag_2_S-MIP-TiO_2_ as a catalyst but also showed that Ag_2_S-MIP-TiO_2_ was chemically stable.

In conclusion, a nanocomposite of Ag_2_S-MIP-TiO_2_ was synthesized using the sol-gel technique. The photocatalyst was observed to be anatase with good crystallinity and showed obvious quantum size effect. The Ag_2_S-MIP-TiO_2_ composite demonstrated better photocatalytic effect than TiO_2_, as it introduced a new impurity level from Ag_2_S to TiO_2_. The new impurity level reduced the crystal size of TiO_2_, reduced the forbidden bandwidth, and became conducive to the transition of electrons. Hence, the application of Ag_2_S could reduce the energy required for electronic transitions, which led to the improvement of the photocatalytic effect of TiO_2_. The Incorporation of Ag_2_S into MIP-TiO_2_ increased the active binding sites of TiO_2,_ causing an increase in the target pollutant molecules, thereby increasing the photocatalytic activity and selectivity factors. It was observed that the activity of Ag_2_S-MIP-TiO_2_ compared to TiO_2_ increased by about 30% with an improvement in the selectivity factor by 1.73 for MIP-TiO_2_. This study explored the effect of the conductivity of molecularly imprinted polymers on the photocatalytic behavior innovatively, and also Ag_2_S-TiO_2_ composite molecularly imprinted polymer was successfully prepared with high selectivity and high photocatalytic activity. Overall, this study provides a new method of water treatment and for the synthesis of high-efficiency photocatalytic materials.

## Methods

### Preparation of catalysts

**Figure 6:**
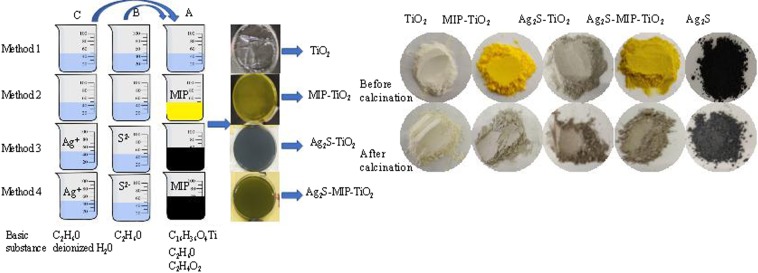
Preparation method of different powder.

As shown in Fig. 6, each powder was synthesized from the liquids using three beakers, A, B, and C. MIP indicates E-pHB (target imprinted molecule) being added. Taking the synthesis method of Ag_2_S-MIP-TiO_2_ as an example, an appropriate 20 ml of n-tetrabutyl titanate, 1.954 g of E-pHB, and 10 ml of glacial acetic acid were dissolved in 40 ml of absolute ethanol and stirred to form solution A. A mixture of 20 ml of absolute ethanol and 0.1311 g of thiourea was stirred uniformly to form solution B. An appropriate amount of deionized water, 20 ml of absolute ethanol, and 0.994 g of silver nitrate was stirred to obtain solution C. The solution C was then charged into the dropping funnel, and mixed with solution A under vigorous stirring in controlled conditions. The solution B was then added dropwise to the solution A under vigorous stirring for 2 h. Following this, the solution was maintained at a constant temperature and kept away from light for 2 days and oven-dried to obtain crystals. The obtained crystals were then ground into a powder using a mortar and hen kept in a muffle furnace for 1 h, as the furnace was heated at 3 °C/min until 200 °C, and followed by 500 °C for 2 h to form the Ag_2_S-MIP-TiO_2_ photocatalyst.

### Characterization of the catalyst

The elemental composition of the sample was analyzed by X-ray photoelectron spectroscopy (XPS, USA, EscaLab 250Xi, Thermo Fisher Scientific). The catalyst was subjected to UV-Visible diffuse reflectance spectroscopy (UV-Vis, Hitachi, model U-3900). The composition of the sample was analyzed by Fourier transform infrared spectrometer (FI-IR, FT-IR650, Shimadzu Corporation, Japan). The crystal phase of the sample was analyzed by Xrertpro X-ray diffractometer (XRD, PANalytical B. V., Netherlands). The morphology and particle size of the samples were observed using scanning electron microscopy (SEM, S4800N, Hitachi, Japan) and transmission electron microscope (TEM, FEI Tecnai G^2^ F20 S-TWIN, USA).

### Photocatalytic experiment

Using the changes in absorbance and concentration, the removal rates could be obtained using Beer-Lambert’s law, and the selectivity factor could be obtained based on the change in the reaction kinetic parameters. 3 g/L of TiO_2_, MIP-TiO_2_, Ag_2_S-TiO_2_, and Ag_2_S-MIP-TiO_2_ were added to 70 ml E-pHB (10 mg/L) and 70 ml phenol solution (10 mg/L) in quartz glass test tubes in sequence. The reaction solution was irradiated with a 500 W mercury lamp which was used as an ultraviolet light source. The sample was taken at intervals of 30 min. After the solution was centrifuged for 10 min, the supernatant was taken in a clean and dry quartz cuvette, and the absorbance of the supernatant was measured using UV-Vis spectrophotometer. The absorbance of E-pHB and phenol was measured at 256 nm and 213 nm, respectively.

### Adsorption performance and conductivity study

Isothermal adsorption experiments were carried out at 30 °C and a stirring rate of 150 r/min by adding respectively 3 g/L of TiO_2_ and Ag_2_S-MIP-TiO_2_ to 70 ml E-pHB solution (10 mg/L). TiO_2_ and Ag_2_S-TiO_2_ were added to E-pHB solution in the same way as above. After mixing evenly, the photocatalytic reaction was carried out under ultraviolet light. The TDS value of the reaction solution was measured by TDS pen at intervals of 30 min and converted into conductivity. The changes in the conductivity of the reaction solution were investigated during the photocatalytic reaction.

### Recyclability study

To carry out ultraviolet photocatalytic reaction, an appropriate amount of Ag_2_S-MIP-TiO_2_ and TiO_2_ powder were added to E-pHB solution in the same method as mentioned above. After the reaction, the waste liquid was centrifuged for 10 min, and the supernatant was discarded. The obtained powder was washed three times each with absolute ethanol and deionized water. The turbid bottom liquid was poured into a cuvette and placed in an oven at 120 °C for 2 h. The recovered powder was then added to a quartz glass tube in the same amount, 3 g/L, to carry out the photocatalytic experiment, and the absorbance was measured twice an hour at the intervals of 30 min.

## Data Availability

The datasets generated the current study are available from the corresponding author on reasonable request.
